# 2-Year-Olds Learning From 2D Media With and Without Parental Support: Comparing Two Forms of Joint Media Engagement With Passive Viewing and Learning From 3D

**DOI:** 10.3389/fpsyg.2020.576940

**Published:** 2021-01-25

**Authors:** Mikael Heimann, Louise Hedendahl, Elida Ottmer, Thorsten Kolling, Felix-Sebastian Koch, Ulrika Birberg Thornberg, Annette Sundqvist

**Affiliations:** ^1^ Infant and Child Lab, Department of Behavioral Sciences and Learning, Linköping University, Linköping, Sweden; ^2^ Department of Psychology, University of Siegen, Siegen, Germany

**Keywords:** digital media, joint media engagement, learning, memory, deferred imitation

## Abstract

The study investigates to what degree two different joint media engagement (JME) strategies affect children’s learning from two-dimensional (2D)-media. More specifically, we expected an instructed JME strategy to be more effective than a spontaneous, non-instructed, JME strategy. Thirty-five 2-year old children saw a short video on a tablet demonstrating memory tasks together with a parent. The parents were randomized into two groups: One group (*N* = 17) was instructed to help their child by describing the actions they saw on the video while the other group (*N* = 18) received no specific instruction besides “do as you usually do.” The parents in the instructed group used significantly more words and verbs when supporting their child but both groups of children did equally well on the memory test. In a second step, we compared the performance of the two JME groups with an opportunistic comparison group (*N* = 95) tested with half of the memory tasks live and half of the tasks on 2D without any JME support. Results showed that the JME intervention groups received significantly higher recall scores than the no JME 2D comparison group. In contrast, the three-dimensional (3D) comparison group outperformed both JME groups. In sum, our findings suggest that JME as implemented here is more effective in promoting learning than a no JME 2D demonstration but less so than the standard 3D presentation of the tasks.

## Introduction

Over the last decades, digital media has become seamlessly integrated in the life of families, a societal change that impacts children’s early experiences. The studies conducted so far tell us that young children, especially if 36 months or younger, seem to have a harder time learning from media than from real life events (Barr, 2010; Barr and Linebarger Nichols, 2017; Strouse and Samson, 2021) but also that excessive screen time at an early age might influence a child’s development negatively (Madigan et al., 2019). However, research also suggests that how parents interact with their children is important for language and cognitive development (Barr, 2019). Although parents own use of technology might interfere with children’s learning by limiting parent-child interactions (McDaniel and Radesky, 2018; Sundqvist et al., 2020), other studies suggest that parents can facilitate children’s learning if they take an active and supportive part in their child’s use of digital media (Zack and Barr, 2016; Barr, 2019; Padilla-Walker et al., 2020). The idea that parental support might ameliorate learning from digital media is the basis of this study in which parents were instructed to employ one of two different interactive strategies in order to support their 2-year-old child’s learning from information provided on a tablet.

A specific form of how parents may support learning from digital media is when parents view media content together with their child in order to support the child’s learning and/or understanding of the content at hand (e.g., Courage, 2017; Padilla-Walker et al., 2020). Although previously labeled co-viewing (Ewin et al., 2020), a more precise, up-to-date, and informative label is joint media engagement (JME) which, as Barr (2019, p. 343) states, “occurs when people interact around media together to scaffold learning.” Exactly when and how JME is effective is, however, not clear. According to Ewin et al.’s recent review, it is relatively common for parents to attempt some kind of JME strategy such as prompting, cognitive scaffolding, or various dialogical strategies. They further suggest that many parents seem to transfer their knowledge on how to deal with traditional TV-viewing to the new digital media environment. The successfulness of these strategies depends, however, on factors such as parental skills, educational level, socio-economic status (SES), and ethnicity. Another factor that Ewin et al. (2020) brings up is that sometimes the media in itself hinder the parent’s attempt to create a JME situation when watching together with a young child. This is because the content sometimes creates such a heightened engagement that it becomes difficult for a parent to “break in” in order to create a dialog. Although comprehensive, the review also makes it obvious that much research is still lacking, the papers covered in the review are unevenly spread both culturally and geographically. About half of the studies comes from North America, about a fifth from Europe and only one single study represents Scandinavia (Norway).

The current study investigates if a specific JME instruction to parents leads to better learning from two-dimensional (2D)-media than when parents are allowed to choose spontaneously how to interact with their child as measured by how much the children remember of a video demonstration of a deferred imitation test presented on a tablet. Deferred imitation (DI) requires forming, storing, and later retrieving information of an observed behavior and is often described as a pre-verbal measure of episodic declarative memory (e.g., Meltzoff, 1988; Heimann and Meltzoff, 1996; Jones and Herbert, 2006; Kolling et al., 2010; Lukowski and Bauer, 2014). Today, DI is an established method to study memory and learning in children with no or very limited verbal ability (Meltzoff, 1988; Barr et al., 1996).

Deferred imitation has been successfully used to examine how children acquire and remember knowledge from screens (e.g., Barr and Wyss, 2008; Myers et al., 2017). Research has shown that toddlers often have a slower learning curve when learning from digital media than from real events or actions. In addition, they also show difficulty to transfer what they have learned from 2D to three-dimensional (3D) or the other way around, a phenomenon referred to as a *transfer* or *video deficit* (Kirkorian, 2018; Barr, 2019; Strouse and Samson, 2021) usually assumed to be most evident among children younger than 3 years (Barr and Brito, 2014; Moser et al., 2015; Courage, 2017; Barr, 2019). In a review covering the video deficit among children 0–6 years old, Strouse and Samson (2021) conclude that the effect seems to decrease as children grow older, but it is uncertain exactly when and if it will vanish completely. About two-thirds of the studies in the review represent North America, only 14% is European and only one comes out of Scandinavia (Sweden). For children below 36 months, Strouse and Samson report that the observed averaged effect size (*g*) was about three times larger than for children older than 3. They also report that the effect varies with the domain studied. As example, object retrieval tasks showed the strongest deficit (*g* = −1.00) while studies focusing on imitation displayed an effect that was about half as strong (*g* = −0.58). The deficit can probably be explained, at least partly, by factors restraining young children’s ability to interpret and process information, foremost limitations in their perceptual abilities (Barr, 2019), as well as a lack of memory flexibility (Barr and Brito, 2014). In addition, young children’s lack of symbolic understanding is most likely also part of the explanation for the transfer deficit. Problem with symbolic understanding also influence young children’s ability to transfer knowledge from books to the real 3D world (e.g., Simcock et al., 2011; Brito et al., 2012). Pictures are symbolic artifacts and it takes considerable time for a child to understand that an object shown on a video is identical with a real-world object. This has been shown by DeLoache (2004) who also have demonstrated that a shift in symbolic understanding occurs sometime between a child’s second and third birthday. With these limitations in regard, transferring information between poorly matching contexts such as from screen to reality constitutes a challenge for young children.

The transfer deficit effect does not indicate that it is impossible for young children to generalize learning from screens (e.g., Barr, 2010; Moser et al., 2015; Kirkorian, 2018); only that it is a challenge. Further, research also suggest that the effect can be decreased by various JME strategies such as scaffolding, using verbal or visual cues, social interaction, or repetitions (e.g., Barr, 2010, 2019; Lauricella et al., 2016). Visual cues can enhance the degree of matching between contexts, thereby reducing the importance of perceptual limitations (Barr and Brito, 2014), and verbal cues can support learning either by being embedded into the media content itself, or received from a present person (e.g., Strouse et al., 2018).

As perceptual skills and memory flexibility develop with age, lack of symbolic understanding is thought to become a greater obstacle for successful transfer in older toddlers (Barr, 2013; Courage, 2017). Generalizing learning from a tablet, for example, to real life demands that the child understands that the tablet is not just an object in itself, but also entails symbolic representation of other objects. A task that is especially difficult for young children (Barr, 2013). Related to this, Strouse and Ganea (2017) found that the transfer deficit effect was less evident in children aged 17–30 months who had more variable experiences of media, as opposed to peers whose only experiences of screens came from watching videos. The authors argue that children who use media for multiple purposes have an easier time understanding the relation between on-screen-events and real life (Strouse and Ganea, 2017), which is in accordance with Barr’s (2013) reasoning on the importance of symbolic understanding.

In addition to the factors discussed above, lack of social interaction has been suggested as a possible explanation for the transfer deficit effect (Troseth et al., 2006). With increasing age, children start relying more on social cues for learning new information (Golinkoff and Hirsh-Pasek, 2006). Unlike real life situations, media content often lacks socially contingent cues, such as eye contact and gestural communication, which help children understand that the information presented is reliable and relevant.

Joint media engagement is a form of socially interactive scaffolding that has been suggested to be effective as a way to counteract the transfer deficit (Courage, 2017; Barr, 2019). It is argued that if adults (read parents) participate when young children use media, they can help them pay attention and make sense of the content and thereby support learning (Barr et al., 2008; Strouse and Troseth, 2014; American Academy of Pediatrics, 2016; Courage, 2017; Samudra et al., 2020). As an example, Strouse et al. (2018) found that 30-month-old children learned more new words when watching a video together with a parent modeling, as opposed to watching the video alone. In contrast to this, Barr and Wyss (2008) found that co-viewing with a physically present person may not be necessary. In their study, 24-month-olds imitated equally well whether they got scripted verbal cues from a parent that was present or from a prerecorded voice. However, since the parents in Barr and Wyss’ study had to follow a script, they examined the mere presence of a parent rather than the naturally occurring parent-child-interaction, which could be of particular importance for learning.

Exactly how and when JME is beneficial is not entirely clarified but taken together, previous research (e.g., Barr, 2019; Ewin et al., 2020) does suggest that socially contingent interactions and verbal cues help young children learn from digital media. We therefore suspect that experiencing JME with a socially responsive adult would compensate for the lack of social contingency in media content, especially for the 2-year old ager group being in focus for this study. In order to investigate this, we initially formulated one key question: (1) To what degree does an instructed verbal strategy of parental JME support children’s learning from video compared to a freer non-instructed JME strategy? In order to answer this question an intervention was created. As a second step, we also wanted to gain information on two additional questions: (2) Does JME result in better learning compared to co-viewing together with a parent who is passive and not using JME at all, and (3) does a live presentation support learning better than all or some of the three groups learning from 2D (instructed JME, spontaneous JME, or no JME)? These two questions became possible to address since we were able to use children participating in a separate study on media and learning as an opportunistic comparison group.

More specifically, in relation to our first question, we hypothesized that children viewing a video of a memory test together with a parent who had been instructed to support attention for learning would remember more actions than children having viewed the video with a parent who had received no specific instructions on how to interact with the child. Further, we were able to draw on an opportunistic comparison group that performed half the tasks after 2D presentations and the other half after a live (3D) presentation. Children in the comparison group watched a 2D presentation of some of the memory test items passively, that is without any verbal support from the parent. Regarding research question two, we expected both JME groups to perform better than children in our opportunistic comparison group when compared with the tasks that were presented in 2D. We were more uncertain whether the two JME groups would perform worse, on par or better than children in the comparison group when compared on the tasks that were presented live (3D), thus this part was exploratory, and no explicit hypothesis was formed.

## Materials and Methods

### Participants

#### JME Intervention Groups

Thirty-five parents (26 mothers, nine fathers) and their 2-year-old child (*M* = 24.2, range 23.1–25.6 months, *SD* = 0.71) participated. The dyads were randomized to either a group that received specific instructions (*N* = 17; eight females) or a group (*N* = 18; eight females) receiving no specific instructions. There were no differences in age or developmental level as measured with Bayley-S (*M* = 108; range 94–123; Bayley, 2006), due to gender or intervention assignment. The parents reported that the children were typically developing with no known medical issues. The families were of middle or high SES, 71% of the mothers and 66% had a university degree. All parents were fluent in Swedish.

#### Parent’s Media Proficiency in the JME Groups

A majority of the participants (58.7% in the instructed group and 61.1% in spontaneous JME group) used a tablet daily or several times a week according to the parents. Their experience with smartphones was at the same level (70.6 and 64.7%, respectively). Parents in both groups reported that they regularly viewed screen content together with their child and a majority that they most of the times discussed the media content with their child (70.2% and 72.2 respectively). Eight parents (four in each JME group) stated that they had never used a tablet together with their child.

#### Comparison Group

Data from 95 two-year-old children (44 female) participating in a separate project were used for comparison purposes. Their mean age was 25.5 months (range 24.8–26.4; *SD* = 0.33). There was no difference in age between male and female participants. All children were reported by the parents to display typical development and a majority of the parents held a university degree (83% of the mothers and 66% of the fathers). Swedish was reported as the main language used at home in all families.

#### Parent’s Media Proficiency in the Comparison Group

Digital media was available in all households and used by both parents and children. All families had a smartphone that was also used by all children. Almost all (95%) households had a TV (used by 87% of the children) or a computer (used by 81% of the children). Tablets were used by 81% of both parents and children.

#### Recruitment and Attrition

All participating children were recruited through the Swedish Population Register (SPAR) and an invitation letter was sent to all families with children in the right age range living in the Linköping area. An invitation letter was sent to all families with children in the right age range living in the Linköping area. For the two JME groups participating in the intervention this meant approaching families with a child turning 2-year between January and April 2018 (*N* = 408) while for the study used as an opportunistic comparison group all families with a child turning 9 months between May and September 2017 received a similar invitation (*N* = 1324). Those who expressed an interest (the response rate is usually between 10 and 15%) were contacted by phone and informed about the studies.

For the JME intervention part, the instructed and the non-instructed JME groups, 47 families replied of which 41 expressed a willingness to participate after receiving more detailed information about the study. Of these, three were later excluded due to lack of proficiency in Swedish and three families were lost due to scheduling difficulties. Thus, the final sample to be included consisted of 35 children.

For the comparison group 102 families accepted to be part of a 2-year follow up of which 93 children provided live presentation (3D) data and 86 children 2D no JME data (the parent was told to be silent) on the specific memory test used in this study. The attrition being mainly due to procedural errors, fatigue, scheduling difficulties, or illness.

### Instruments

#### The Frankfurt Imitation Test-24 (FIT-24)

It consists of eight object-based tasks adapted for 2-year-old children in order to measure early declarative like memory through the method of deferred imitation (Kolling et al., 2010; Kolling and Knopf, 2015). That is, the test procedure includes three phases: a demonstration phase, a delay of about 30 min (range 20–40 min across all groups in this study) and a recall phase. During the demonstration phase, the children are only allowed to watch as the experimenter demonstrates the actions; it is not until the recall phase that the participants were allowed to manipulate the objects and to produce the target actions.

All demonstrated actions were multi-step sequences, 6 three-step actions, 1 five-step action, and 1 six-step action. An example of a three step-action is the first item, the Gondola. The actions demonstrated were (1) to place a manikin in a plastic gondola, (2) to lean a spoon against the manikin, and (3) to move the gondola back and forth. The second item is an example of tasks requiring more than three steps. The target actions presented for this task, were (1) to pull a blue sheet from of a container, (2) to open the container, (3) to take out a different manikin, (4) to bend the manikin’s legs, and (5) to place the manikin in the boat. See Table 1 for descriptions of all items. The original FIT-24 was developed for live demonstrations and each task should be demonstrated twice according to the manual (Kolling and Knopf, unpublished) and the score (0–29) depends on the number of target actions produced by the child.

**Table 1:** Description of the tasks in the Frankfurt Imitation Test (FIT) and their inclusiveness in the different groups [adapted from Kolling and Knopf, (2015, pp. 366–368)].

Task	Description	Steps	JME^1^	Comparison^2^	
			2D	2D	3D
1.Gondola	Place a manikin in plastic gondola, lean a spoon against the manikin, and move the gondola back and forth	3	Yes	No	Yes
2.Boat and box	Pull a blue sheet from of a container, open container, take out a different manikin, bend its legs, and place the manikin in the boat	5	Yes	No	Yes
3.Frog	Lean a board toward stand, frog jumps onto stand, slides down	3	Yes	No	Yes
4.Ball with eyes	Find slit in a ball, insert eyes. and let ball jump up and down	3	Yes	Yes	No
5. Turtle	Click a cone on ball, place both on turtle, and lift up the turtle	3	Yes	No	Yes
6. Bunny	Attach yellow pillow, green square pillow, and a triangular pink pillow on a bunny	3	Yes	Yes	No
7. Box	Place ring on a hook, spin the ring, and open drawer (bird appears)	3	Yes	Yes	No
8. Magnetic plate	Turn the plate, put red button on top, a yellow button below the red, a black button below the yellow, stick croissant on plate, roll plate	6	Yes	Yes	No

1 Two active joint media engagement intervention groups (*N* = 17 and 18, a between subjects design; see text for details).

2 A within-subjects design: 2D = presentation by video (*N* = 86); 3D = Live presentation of tasks according to the manual (*N* = 93).

#### Questionnaire

A brief questionnaire-based interview focusing on the child’s developmental history and media proficiency was conducted with the parents in the two JME groups before the experiment. The media questions focused on smartphone use, tablet use, JME, and how often the parents usually talk with their child about media content. The parents in the comparison group answered similar questions through a web based survey administered through Qualtrics.

### Procedure

#### JME Intervention Groups

**Figure 1:**
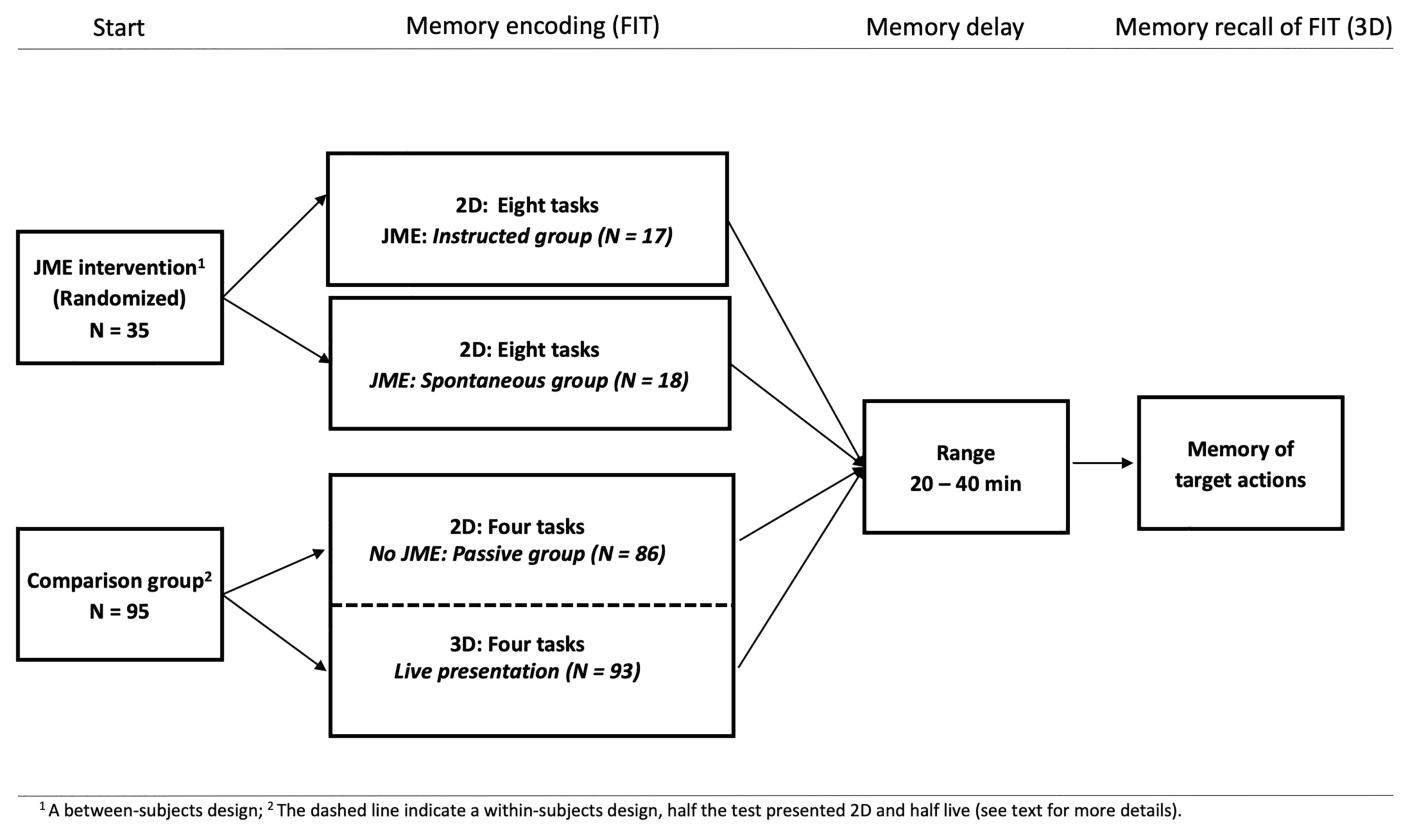
The figure displays the overall design of the procedure and the groups included: The two JME intervention groups watching the complete Frankfurt Imitation Test (FIT) on a tablet and the children in a separate study used for comparison purposes being presented with half the same test (four items) either passively on a computer screen (2D) or live (3D).

All children in the two JME intervention groups were observed once at the Baby-and Child Lab at Linköping university, Sweden, and each session took 1 h (*M* = 59.74 min; *SD* = 4.86). Two graduate students tested all children (17 and 18 children each, randomized). Before the session began the parents were informed of the procedure and signed an informed consent form. For a complete overview of the procedure, see Figure 1.

The parents were informed that they would view a video on a tablet that showed a person demonstrating actions with different objects. The instruction was manualized and parents in both groups were initially given the following information: “You will now watch a short video made up of several short video clips together with your child. In the video you will see a person show several different toys and what you can do with them.” After this each group received specific instructions where after the experimenter asked if the parents had any questions: “If you do, it’s best if you ask now, so you can focus entirely on the film later.” The specific instructions varied depending on their group assignment: Parents belonging to the *instructed joint media engagement group* were told to verbalize what was presented in the video clips. That is, the parent was expected to describe to the child the actions presented in order to support attention and thus strengthen the child’s memory of the target actions. The specific instructions read to the parents in the instructed group were: “I want you to put into words what happens in the various clips so that your child remembers as much as possible. You can tell what’s going on, step by step. For example, like this, ‘look, now she takes the blanket and puts it over the doll’. It might feel a bit difficult, but just try to talk to your child about what the person in the video is doing.” In contrast, the parents in the *spontaneous joint media engagement group* were only told to watch the video together with their child as they would have done if they had been at home. The specific text read to the parents in this group was: “I want the two of you watch the film together, as you would if you had watched it together at home. It may feel a little strange to do in this environment, but just try to do as you would have done at home.”

During the video demonstration of the FIT-24, the child sat on the floor together with the parent. Most children sat on their parent’s lap.

#### Presentation of Target Actions for the JME Intervention Groups

For the purpose of this study, the demonstration phase of all eight FIT-24 tasks were presented in 2D-format on a tablet: Each parent-child dyad watched an 8 min 46 s long video showing a woman demonstrating the eight tasks. In order to ameliorate for a possible transfer deficit, the video contained three demonstrations of each task. This decision was based on research suggesting that an extra repetition sometimes do counter the learning problems associated with the transfer deficit (e.g., Barr, 2010). In addition, we also based our decision on a previous study from our own lab that investigated how infants attend to 2D presentations which revealed a dynamic change in “the distribution of infants’ attention to a presenter’s face and the action she performs” (Koch et al., 2018, p. 196). Great care was also taken to make sure that video-recordings would be social in a way that the presenter greeted the child and looked straight into the camera before presenting the tasks (i.e., at the child). The presenter’s verbal utterances exhibited both interest and excitement in the actions she was about to present. As the presenter demonstrated the action her gaze shifted toward the objects. While performing the action she was quiet, and immediately after she looked back into the camera and expressed a happy and joyful face. Her verbal comments were of a general nature and the presenter did not address the specific actions. Any verbal cues specific to the content of the videos were produced solely by the parents. In order to alleviate any problems in perceptual matching, the objects the presenter used in the video were identical to the ones the child would handle during the recall phase. The interval (*M* = 27.2 min; *SD* = 4.22) between watching the video and the recall phase was used to give the child a brief paus and to administer three subscales (cognition, expressive and receptive communication) from Bayley-S (Bayley, 2006).

#### Comparison Group

The children in the comparison group participated in a larger longitudinal study on early memory, media and language conducted at the same lab (see Figure 1). Their visit was divided into two sections (each 45–60 min long) with a 20–30 min pause in-between. The 2D part was conducted as part of the first section since piloting had shown that this part was more taxing for the children. The 3D presentations of the tasks were administered after the pause when the children had regained both motivation and energy. Beside the FIT-24 test in focus here several additional measures not relevant for the present study were presented during the visit (e.g., measures of language, implicit memory, communication and social skills).

#### Presentation of Target Actions for the Comparison Group

The children used for comparison purposes participated in a comprehensive study on learning and media where both 3D and 2D tasks were used and FIT was included as memory test. Since it was both methodological and theoretically impossible to administer all eight tasks included in FIT both as a real life administration (3D) and as 2D on a computer screen the test was split so that four items were used in each condition. The final split, based on extensive pretesting, created two sets of four items with similar levels of difficulty. Each set included three 3-step tasks and one 5-or 6-step task. During the interval (*M* = 34.5 min; *SD* = 8.7) between presentation and recall the children participated in other tests such as an implicit memory test for the no JME 2D presentation and a socio-communicative test for the live presentation. A brief pause was also included. Based on piloting and in order to limit attrition due to fatigue, the no JME 2D was presented early during the visit and the 3D live presentation after the pause.

For the passive no JME 2D viewing comparison, the tasks included three three-step tasks (Ball with eye, Bunny and Box) and one 6-step task (Magnetic plate) that were all presented as video clips on a computer screen using the exact same recordings as shown on the tablet for the JME intervention groups. Similar to the 2D procedure used for the instructed and spontaneous JME groups three demonstrations of each task were used in order to minimize the transfer deficit effect. The children sat in their parents’ lap in front of a computer screen, approximately 60 cm away from the child’s face, silently watching the videos. The parents were instructed to be silent and not to interact with their child or to comment on what was shown although they could verbally support their child’s attention by for instance saying “look at that.” However, they were also told that brief gazes away were unproblematic and did not matter. A curtain that separated the experimenter from the parent and infant was closed before starting the calibration procedure that always preceded the presentation of the 2D tasks.

For the 3D presentation, three 3-step tasks (Gondola, Frog and Turtle) and one 5-step task (Boat and box) were administered live in a separate room at the lab following the procedure described in the original publication (see Kolling et al., 2010). This entailed that each task was presented only twice.

### Statistical Analysis

The analysis was conducted in two steps. First the two JME intervention groups are analyzed in relation to how successful the intervention was (e.g., the parents verbal behavior), if the memory recall score differed between the groups and if other factors affected the findings (e.g., the child’s attention to the tasks). In this first step, the score from FIT is based on all eight items. The second step entailed comparing the FIT recall score observed for the JME intervention groups with the result observed for the children used for comparison purposes. In this analysis only half of the FIT tasks are used in each comparison. The statistical methods for in-between group comparisons are Student’s *t*-test when equal sample size and variances are observed and Welch’s *t*-test when sample sizes and/or the variances differ (see Delacre et al., 2017). The statistics were computed in SPSS version 26 or Jamovi version 1.2.27.0. An *α*-level of 0.05 is used throughout. Effect sizes are reported as Cohen’s *d* when groups are equally large and as Hedge’s g when comparing groups that differ in size.

#### Reliability

The FIT-24 deferred imitation test was coded according to the German manual (Kolling and Knopf, unpublished) and reliability was checked by two of the authors (L. H, and E. O.) who independently recoded a random selection of 25% of the videos resulting in an average reliability score of 0.83 (Pearson’s *r*). The lowest reliability coefficient was noted for item 1 (*r* = 0.79) and the highest for item 2 (*r* = 1.00). For all other items the observed coefficient varied between 0.80 and 0.96. According to Rosso (2003) an r of 0.70 should be considered the lowest acceptable result when using Pearson r. As a rule of thumb, an r >0.80 is viewed as good while a coefficient >0.90 indicates excellent reliability.

### Ethical Statement

The study was approved by the Regional Ethics Review Board in Linköping (no. 2016/490-31).

## Results

### A. The Intervention: Comparing Instructed and Non-Instructed JME

There were no differences in session length between the conditions, male and female participants or between mother-infant and father-infant dyads on any of the measures (*ps* > 0.501). Thus, neither session length, child or parent gender are analyzed further. In addition, gaze away that is how many times a child looked away from the screen while a task was presented was coded as a proxy for attention. As shown in Table 2, the observed frequencies did not differ between the two groups in how many times they looked away from screen, *t*(25.8) = 0.47, *p* = 0.64, *d* = −0.08, equal variances not assumed.

**Table 2:** Parent verbal interaction, session length, and memory result for the two joint media engagement (JME) intervention groups.

	Instructed JME *N* = 17		Spontaneous JME *N* = 18			
	*M*	*SD*	*M*	*SD*	*P* <	*d* =
Words used (freq)	561.65	224.55	177.41	161.46	0.001	1.93
Verbs used (freq)	51.82	22.82	10.59	11.35	0.001	2.26
Gaze away (freq)	9.76	7.09	8.82	4.14	ns	−0.16
Memory delay (min)	26.24	3.91	28.11	4.40	ns	−0.44
Memory recall score	15.94	5.55	16.33	3.77	ns	−0.08

#### Verbal Scaffolding

The intervention strongly influenced how the parents interacted verbally with their children (see Table 2). The groups differed significantly with respect to the total number of words the parents used during the video demonstration of the tasks, *t*(32) = 5.73, *p* < 0.001, *d* = 1.96, and also with respect to the number of verbs used, *t*(23.46) = 6.67, *p* < 0.001, *d* = 2.29, equal variances not assumed. This difference between the groups reflect the fact that the parents in the instructed JME group used on average approximately three times the number of words [*M* = 561.65; 95% CI (446.20–667.10)] than the parents in the spontaneous group [*M* = 177.41; 95% CI (94.40–260.43)] while watching the video together with their child. A similar strong difference between the groups was also noted when comparing the parents’ use of verbs. In spite of the observed difference in how much the parent’s spoke, no parent was completely silent but three parents used less than 50 words.

#### JME and Learning From 2D

There was no difference between the two JME groups in learning as evident from the obtained memory recall score, *t*(33) = −0.246; *p* = 0.81 (Table 2). In other words, there was no memory advantage for the children to the parents having received instruction to verbally support their child which was contradictory to our expectations.

### B. Comparing the JME Intervention Groups With No JME 2D and Standard 3D Presentations

Since the children in both JME groups performed equally well on the deferred imitation memory test (FIT-24) their data were collapsed and thereafter compared with the results from the comparison group.

#### 2D With JME vs. Passive 2D Viewing

Since only half of the tasks in the FIT-24 memory test was used for the passive 2D presentation the comparison group saw, the mean score for the two JME groups was based on the same four items (see Table 3). This comparison revealed that the children in the JME groups (*N* = 31) received a significantly higher memory score than the children (*N* = 86) having viewed exactly the same 2D video presentations passively on a computer screen, Welch’s *t*(94.55) = 2.65, *p* = 0.009, equal variances not assumed; Hedges *g* = 0.42.

**Table 3:** Recall memory scores on the Frankfurt Imitation Test (FIT) for the JME intervention groups and the opportunistic comparison group being tested with half the items 3D and half 2D.

FIT		2D JME			2D no JME			3D Comparison				Max	*N*	*M*	*SD*	*N*	*M*	*SD*	*N*	*M*	*SD*	*P* ^2^ =
Items A^1^	15	31	9.52	1.59	86	8.41	2.83	-	-	-	0.012
Items B^1^	14	35	7.60	2.95	-	-	-	93	9.57	2.55	0.001

1 Items A = tasks number 1, 2, 3, and 5; Items B = tasks number 4, 6, 7, and 8. See Table 1 for description of tasks and text for details.

2 Welch’s *t*-test due to unequal N (between-subjects analysis).

#### 2D With JME vs. Live Presentation (3D)

In a similar fashion as above, the results for the JME 2D groups were compared with the 3D presentation used for the comparison group. The 3D procedure used was closely aligned with the procedure outlined in the manual and in the German standardization of the test (Kolling and Knopf, 2015). The results from the JME groups were based on the same four items that had been used with the comparison group (see Table 3). The analysis revealed that the comparison group having seen the target actions live (*N* = 93) displayed a better memory of the target actions than the children in the JME group, Welch’s *t*(54.2) = −3.49, *p* < 0.001, equal variances not assumed; Hedges *g* = −0.74.

## Discussion

This study compared how two forms of joint media engagement (JME) might support two-year old children’s long-term memory after watching a 2D demonstration of eight different actions on a tablet. All children saw the video together with one of their parents and learning was measured by how many actions from the presented tasks the children recalled after a delay of approximately 30 min. The children were randomized to either an instructed or a spontaneous JME group. Parents in the instructed JME group were explicitly told to verbally support their child’s learning while the parents in the spontaneous group were only instructed to support their child as they would ordinarily do if watching at home. The children’s learning and memory performance was evaluated first by comparing how the two JME groups performed, second by comparing the JME groups with 2D learning without JME, and third by comparing if 2D JME differed from 3D (live) learning.

In response to our primary research question, to what degree an instructed JME strategy would support children’s learning from 2D compared to a freer and non-instructed JME strategy, we found that the two strategies, as measured by children’s recall of target actions, did not differ. However, the intervention was successful to the degree that parents in the two groups differed significantly in how much they talked with their child. Parents in having received specific instruction regarding their JME strategy used three times more words and five times the number of verbs than the parents receiving no specific instructions but this difference in verbal scaffolding did not affect the children’s recall. Our hypothesis that instructed JME would support child’s learning from the video more than the spontaneous group was not confirmed. The parents in the spontaneous group seems to have been verbal enough since the increased verbal activity observed for the parents in the instructed group did not promote better learning.

The two JME groups did not differ on overall memory recall and also not on gaze away, a proxy for attention. This suggests that both JME strategies are equally potent in supporting learning from 2D media and that this effect is not due to the amount of verbal support given. Attention might in fact be the process that drives learning from educational media as suggested by Samudra et al. (2020) and the fact that the JME groups did not differ on our attention measure could thus be the main reason why we did not observe any difference in learning between the JME intervention groups. One might speculate that parents in both groups already were well acquainted with their child’s learning strategies, they knew how to tune in to their child’s state of mind and they were, therefore, successful in supporting both learning and attention. Future researchers need to study these aspects in more detail as well as the overall emotional climate between the JME partners in order to better understand exactly what an optimal JME interaction should be built upon. According to Padilla-Walker et al. (2020) it is furthermore important to code for both positive and negative behaviors when analyzing JME in detail. They especially underscore the importance of including codes such as positive and negative parental empathic concern.

The second research question focused on if learning with JME supported children’s learning better than passively co-viewing together with a parent using no JME strategies. This was confirmed as we found that the employed JME strategies promoted better support for learning than no JME 2D viewing. Children having viewed a 2D presentation of the tasks together with a parent using either of our two JME intervention strategies displayed significantly better recall scores than the children in the comparison group having viewed the video together with a parent who was instructed to be silent. The observed effect size was close to medium. Thus, it seems that the instructed and the spontaneous JME strategies, as used in the current study, are equally potent in counter acting the so-called transfer deficit (Barr, 2019). It is worth remembering that the videos used were identical in all three 2D presentations, instructed JME, spontaneous JME, and the comparison group receiving no JME. The video was produced with the goal to make learning from a screen easier than usually observed for 2D presentations. The tempo, the gaze and the gaze shifts of the presenter in the video as well as the change from talking to presenting the actions were carefully timed and edited such that the actions would be salient to the child. Thus, the observed difference in learning between the three 2D presentations is not due to how the tasks were shown on the screen since all children saw the same videos. A possible interpretation, in our view, is that the observed effect rests on the JME strategies used by the parents.

In order to address our third question, if a live presentation support learning better than learning from 2D, we compared the collapsed performance of the two JME intervention groups with all 93 children in the comparison group having been tested live with the Frankfurt Imitation Test (Kolling and Knopf, unpublished). The result showed that the JME procedures used by the parents in our study, although helpful, failed to completely ameliorate the transfer deficit effect: Children in the comparison group being tested live (3D) performed significantly better than the JME groups, something which is evident by the relatively high effect size observed which was close to being judged as strong (Hedges *g* = −0.74). This effect size between 2D and 3D learning is within the expected range for a study that uses an imitation paradigm when studying learning and the transfer/video deficit. Strouse and Samson (2021), in their meta-analysis of the video deficit effect, found that the average weighted effect size for imitation studies was −0.58 with a 95% CI (−0.76, −0.41). Moreover, the 3D presentation in the present study resulted in a higher mean score than the 2D JME strategies in spite of the fact that the tablet version of the test included three presentations of each task instead of two as prescribed in the manual for live testing and used here. A procedure that we had expected would have boosted learning from 2D.

Overall, we conclude that both 2D JME strategies employed in our study were equally potent to improve the children’s recall beyond what passive 2D viewing without JME would entail. This is an important observation. However, of equal interest is our finding that the standard 3D presentation actually resulted in a significantly better recall than the 2D presentations used in the study (the collapsed 2D JME groups as well as the no JME comparison group). It surprised us that the results from the JME strategies employed did not differ as measured by the observed memory scores. One possible reason for this might be that the parents in both groups were well educated representing medium to high SES and had already developed workable JME strategies. A majority of the parents in both groups revealed that they often watched digital media together with their child. It is thus probable that they had developed good enough JME strategies in order to support their child’s attention while learning from media. It might be that the children in the two JME groups had had a variable media experience and thus were more open to learn from media than many of their peers since children’s experience with symbolic media, being it traditional 2D books or 2D digital media, affects “their likelihood of transferring information to the real world” (Strouse and Ganea, 2017, p. 139). However, we still lack detailed information as to why the strategies were equally potent in supporting learning is spite of the fact that the parent’s verbal activities differed strongly between the groups. Factors like joint attention, tempo or emotional attunement are all aspects that might provide us with more specific answers as to exactly what constitutes the active supportive ingredient parent’s use. Recently Padilla-Walker et al. (2020) highlighted the importance of parental empathic concern for the development of positive JME experiences.

### Limitations

There are some important limitations to take into consideration when evaluating the results. A major limitation is that both the procedure and the sample size differ between the comparison group and the two JME-groups. In addition, the children in the comparison group were on average 1 month older than the children receiving our JME intervention. The benefit of the included comparison group also becomes partly limited since only half of the memory test was presented live and half as 2D without JME. The reason behind this, as stated earlier, was that the children in the comparison group needed to encounter unknown tasks both for the 3D and 2D presentation. Even though the selection of items for 2D or 3D was decided after pilot observations in order to equalize the difficulty of the tasks selected, any attempt to replicate the findings should use the complete test also for the comparison group. Furthermore, the fact that both an in-between and a within-subjects design were used also limits the lessons possible to draw from the study. The small sample size included in the two JME groups is an added limitation since this could have affected the statistical power available for the analysis. However, the almost identical performance of the two groups makes it unlikely that a larger N would have changed the result. This is further underscored by a mean difference between the two groups in observed memory recall score not larger than 0.39 and an obtained effect size of only −0.08. Such a low effect size suggests that the two JME strategies had an almost identical effect on the children’s learning. It is also worth remembering that the effect size observed for the comparison between 2D no JME and the 3D presentation was in line with what other studies have reported.

The study as a whole also suffers from the fact that all participating families were well-educated and represented middle or high SES. Thus, we do not know to what degree the observed findings are generalizable to the society as a whole. Finally, the fact that the study were carried out in a lab and not at home might also affect the generalizability of the findings.

### Conclusion

We conclude that JME can be effective in promoting learning from 2D. Children receiving either one of the two JME strategies employed performed better than children receiving a 2D presentation without JME support. What exactly entails a good JME strategy needs further studies since the two strategies employed here did not affect children’s learning differently. In spite of the observation that the parents’ verbal activity differed significantly between the two strategies. However, this could be rephrased as indicating that even the minor levels of JME used in our intervention groups have a positive effect on children’s media learning. Finally, our findings also suggest that learning from 3D was the most effective way of promoting learning. In other words, our JME strategies reduced the transfer deficit but could not wipe it out.

## Data Availability Statement

The raw data supporting the conclusions of this article will be made available by the authors, without undue reservation.

## Ethics Statement

The studies involving human participants were reviewed and approved by The Ethical Review Board, Linköping University, Sweden. Written informed consent to participate in this study was provided by the participants’ legal guardian/next of kin.

## Author Contributions

The study was conceived by MH, AS, LH, EO, and F-SK. Data were collected by LH, EO, AS, F-SK, and UB, and analyzed by MH. The first draft of the manuscript was written by MH, LH, and EO. MH, AS, LH, EO, F-SK, and UB contributed to the final version. All authors contributed to the article and approved the submitted version.

### Conflict of Interest

The authors declare that the research was conducted in the absence of any commercial or financial relationships that could be construed as a potential conflict of interest.
